# Sociodemographic Profile Associated with Congenital Heart Disease among Infants Less than 1 Year Old

**DOI:** 10.21203/rs.3.rs-2548938/v1

**Published:** 2023-02-08

**Authors:** Michelle Tran, Anna Miner, Carlin Merkel, Kenton Sakurai, Jessica Woon, John Ayala, Jennifer Nguyen, Jeraldine Lopez, Jodie K. Votava-Smith, Nhu N. Tran

**Affiliations:** University of Southern California/Children’s Hospital of Los Angeles; University of Southern California/Children’s Hospital of Los Angeles; University of Southern California/Children’s Hospital of Los Angeles; University of Southern California/Children’s Hospital of Los Angeles; University of Southern California/Children’s Hospital of Los Angeles; Cardiac Registry Support; University of Southern California/Children’s Hospital of Los Angeles; Children’s Hospital of Los Angeles; University of Southern California/Children’s Hospital of Los Angeles; University of Southern California/Children’s Hospital of Los Angeles

**Keywords:** congenital heart defects, sociodemographic, infants, maternal education, social determinants of health

## Abstract

**Background:**

Congenital heart disease (CHD) affects thousands of newborns each year in the United States (US). Infants born with CHD have an increased risk of adverse health outcomes compared to healthy infants. These outcomes include, but are not limited to, neurodevelopmental, surgical, and mortality-related outcomes. Previous US-based research has explored how sociodemographic factors may impact these health outcomes in infants with CHD; however, their impact on the risk of CHD is unclear. This study aims to explore the sociodemographic profile related to CHD to help address health disparities that arise from race and social determinants of health. Defining the sociodemographic factors associated with CHD will encourage policy change and the implementation of preventative measures.

**Methods:**

This study is a secondary analysis of longitudinally collected data. We compared infants with CHD and healthy controls. We used a questionnaire to collect sociodemographic data. Pearson’s chi-square test/Fisher’s exact tests analyzed the associations among different sociodemographic factors between infants with CHD and healthy controls.

**Results:**

We obtained sociodemographic factors from 30 healthy control infants and 39 infants with CHD. We found a statistically significant difference in maternal education between our 2 groups of infants (p=0.004).

**Conclusion:**

Maternal education is associated with CHD. Future studies are needed to further characterize sociodemographic factors that may predict and impact the risk of CHD and to determine possible interventions that may help decrease health disparities regarding the risk of CHD.

## Introduction

Congenital heart disease (CHD) is one of the most common birth defects in the United States (US), affecting approximately 1% of live births, or 40,000 newborns annually [1]. Infants with CHD often require surgical intervention and may be at an increased risk for adverse outcomes, including not only death but long-term morbidities and adverse neurodevelopmental outcomes, compared to their healthy counterparts [2]. Due to the prevalence of CHD and its impact on both morbidity and mortality among infants, the sociodemographic profile related to CHD and how it may relate to outcomes are important to study [1].

Previous studies have assessed the impact of race, ethnicity, and other sociodemographic factors on outcomes, survival, and mortality in children with CHD [2–4]. Despite an increase in survival rates in the present era for infants with CHD due to improved medicine and surgical interventions, some studies have found that certain sociodemographic characteristics may limit survival rates and can also increase risk for poor CHD outcomes and mortality [4, 5]. A systematic review and meta-analysis showed an association between increased area-based poverty and mortality in infants with CHD [3]. Poverty could potentially compromise survival rates for those with CHD because corrective surgeries, like open-heart surgery, may have higher post-discharge mortality and increased unplanned hospital readmissions [2]. One population-based study demonstrated protective qualities of higher maternal education and private insurance against adverse outcomes in infants with CHD, demonstrating the need to target specific socioeconomic disparities in health [4]. These studies, however, did not investigate the direct effect of sociodemographic factors on the risk of CHD. Thus, most published literature on this topic focuses on the survival and outcomes of infants with CHD after birth and diagnosis.

Among the very few publications that have evaluated the impact of sociodemographic factors on the risk of CHD, findings were inconsistent and varied by country. Studies from Sweden [6] and Canada [5] found that living in a low-income neighborhood is a strong predictor for CHD. Population-based studies conducted in Asia found that low maternal education [7], low maternal income [7], advanced maternal age [8], and history of previous unfavorable fetal outcomes [8] were associated with increased risk for CHD. However, a British population-based study found no evidence for an association between advanced maternal age and the risk of CHD [9]. In addition, studies conducted in Morocco and Iran found no association between previous unfavorable fetal outcomes and the risk of CHD [10, 11]. Previous studies have examined the impact of sociodemographic factors on the risk of CHD in other countries, but to our knowledge, no study has examined the role of sociodemographic factors on the risk of CHD between infants with CHD and healthy controls in the US.

To cover these gaps in knowledge, this study aimed to explore and describe the sociodemographic profile of infants with CHD compared to healthy infants within our research study at Children’s Hospital Los Angeles (CHLA), located within Los Angeles, California (CA), in the United States. We hypothesized that sociodemographic differences exist between infants with CHD and healthy infants. By focusing on key sociodemographic differences that might be linked to the risk of CHD, this study can provide further context for future studies and public health recommendations to help alleviate disparities in the risk of CHD caused by sociodemographic differences.

## Materials & Methods

### Study Design

We compared infants with CHD and healthy controls by conducting a secondary analysis of data collected longitudinally between January 2018 and January 2019 [12].

#### Study Sample

We generated our primary dataset from infants with CHD who were recruited from the following areas of CHLA: the Fetal Maternal Center, the Fetal Cardiology Program, and the Cardiothoracic Intensive Care Unit. We recruited healthy control infants from the Fetal Cardiology Program and the AltaMed newborn clinic within CHLA.

Our original study included infants with CHD who were: (1) ≥37 weeks gestational age at birth and (2) had a documented structural heart defect that required neonatal admission to CHLA for additional monitoring or intervention. Additionally, we included healthy controls who were: (1) ≥37-week gestational age at birth, (2) had no major prenatal, delivery, or postnatal complications, (3) had no congenital abnormalities, and (4) had an uncomplicated neonatal course.

#### Data Collection

We obtained written informed consent from the parents of all participating infants before any study procedures began. We obtained infant demographics and maternal medical history through a questionnaire administered to parents during the infants’ study visits at < 2 weeks of age, 3-months of age, 6-months of age, and at 9-months of age. We used the first completed questionnaire from each infant for statistical analyses.

### Sociodemographic variables

The parents of the infant reported: (1) infant’s sex (male, female), (2) maternal age (< 35, ≥35 years), (3) infant’s race/ethnicity (Caucasian, Hispanic/Latino, Asian, African American, Other), (4) highest level of maternal education completed (≤ 2-year college or technical training, ≥ 4-year college or equivalent), (5) family annual household income (<$35,000, $35,000-$74,999, ≥ $75,000), (6) total number of people in household (≤ 3 people, 4–6 people, ≥ 7 people), (7) primary language spoken at home (English, Spanish), and (8) insurance status (public, private). Initially, the questionnaire had 6 options for parents to select for the highest level of maternal education completed (none, ≤elementary school, high school diploma or GED, 2-year college or technical training, 4-year college or equivalent, postgraduate education) for parents to select; however, based on the distribution of the responses falling within 2 overall groups, we decided to recategorize maternal education into a binary variable (≤ 2-year college or technical training, ≥ 4-year college or equivalent) for analysis purposes.

### Statistical analysis

We performed and summarized descriptive analyses (stratified by infants with CHD and healthy controls) as the frequency with proportion for each sociodemographic variable. Pearson’s chi-square tests evaluated differences and associations between the 2 groups of infants for each sociodemographic variable. Fisher’s Exact tests examined differences and associations between pairs of categorical variables with a subgroup < 5 in a category. We performed all statistical analyses using StataCorp STATA 16.1 (College Station, Texas, US). P-values ≤ 0.05 indicated statistical significance.

## Results

### Participants’ Characteristics

We collected information on infant demographics and maternal medical history on a total of 69 infants (30 healthy control infants, 39 infants with CHD). Among assessments of infants with CHD, we found a high prevalence of the following characteristics: female (56.4%), Hispanic descent (68.4%), maternal age < 35 years (64.1%), ≤ 2-year college or technical training being the highest form of maternal education (75.0%), total household of 4–6 people (72.2%), English being the primary language spoken at home (61.1%), annual household income between $35,000-$74,999, (40.0%), and use of public insurance (61.5%). Among assessments performed on healthy controls, we found a high prevalence of infants with the following attributes: male (53.3%), Hispanic descent (48.3%), maternal age of ≥ 35 years (56.7%), ≥ 4-year college being the highest form of maternal education (60%), total household of 4–6 people (66.7%), English being the primary language spoken at home (73.3%), annual household income of ≥$75,000 (48.3%), and an equal proportion of use of public insurance (50.0%) and private insurance (50.0%). We detailed infants’ characteristics in Table 1. We described the cardiac subtypes among infants with CHD in Table 2.

#### Sociodemographic Differences Between Groups Of Infants

Table 3 presents the relationship between sociodemographic factors and infants with CHD versus healthy controls. We observed a statistically significant difference in levels of maternal education between infants with CHD and healthy controls (p = 0.004).

## Discussion

We found a statistically significant association between maternal education within our infant populations, indicating that levels of maternal education differed between infants with CHD and healthy controls. We found that mothers of healthy controls had higher levels of education compared to mothers of infants with CHD. Results from our analyses align with additional studies in demonstrating that differences in maternal education exist between infants with CHD and healthy infants, suggesting that maternal education may be related to the risk of CHD in the US. Other countries, like China and Canada, also found differences in maternal education between infants with CHD and healthy infants – they found that mothers with higher levels of education (> 12 years) were less likely to have an infant with CHD compared to mothers with lower levels of education (< 12 years) [5, 7]. Our findings elaborated on previous literature by introducing the possibility of maternal education being a surrogate for other sociodemographic differences affecting the risk of CHD, such as diet, access to care, [13] or maternal decision in continuing with the pregnancy [14, 15]. For instance, previous case-control studies conducted in China and Northern Ireland demonstrated that low maternal education was associated with poor diet [13] and lower rate of medication use, such as maternal folic acid supplementation [7, 13] and prenatal vitamins [13]. Folate deficiency during pregnancy can result in birth defects such as neural tube defects, low birth weight, and possibly CHD, as studies have found that mothers who take folic acid supplements are less likely to have an infant with CHD [16]. These outcomes associated low maternal education with impaired fetal development [13]. Maternal education may contribute to the risk of CHD through its interplay with other sociodemographic determinants of health—lower education level can negatively affect prenatal care, leading to poorer diet (defined as folic acid-deficient and containing fewer fruits and vegetables) [7, 8, 13]. With this understanding, it is important to target these interrelated sociodemographic factors directly. An example of an existing program that addresses nutritional issues and risk is the Special Supplemental Nutrition Program for Women, Infants, and Children (WIC) [17]. Our findings present an opportunity to further develop public health and preventative care approaches similar to that of WIC, to address disparities that may lead to an increased likelihood of having an infant with CHD.

Maternal education may also be a surrogate for maternal decision to either continue or terminate a pregnancy with a fetus with CHD. A prospective descriptive study conducted in Israel found that higher education was one of the main factors associated with the decision to terminate a pregnancy after a prenatal diagnosis of fetal CHD [15]. A US-based analysis of 53,000 pregnancies and a retrospective study in Turkey also found similar findings – the rate of terminating a pregnancy after a severe congenital anomaly was diagnosed increased as maternal education increased [14, 18]. These studies associated maternal education to decision-making of terminating a pregnancy when a congenital anomaly diagnosis is present. The association between maternal education and decision-making of either continuing or terminating a pregnancy can be interrelated with many other factors. For example, women with lower levels of education may be unable to take time off from work to utilize prenatal care and therefore not be able to detect a congenital anomaly, if one is present, compared to women with higher levels of education and consequently higher-paying jobs who are able to take time off [19].

Moreover, educational attainment and income are strong predictors of socioeconomic disparity in health outcomes [4]. In this case, we found differences in maternal education within our study between our 2 groups of infants. However, within our sample, our study did not find any statistically significant differences in household income between infants with CHD and healthy controls, in contrast to what we originally hypothesized. A population-based retrospective cohort study conducted in Canada found that infants born to mothers living in low-income neighborhoods were more at risk for developing CHD compared to infants born to mothers living in higher-income neighborhoods [5]. The Canadian study suggests that income contributes to the risk of CHD and that the neighborhoods that mothers live in may impact the likelihood of their infant being born with CHD. Our study did not have comparable findings. This may be due to differences in study populations as Miao’s study cohort included infants from hospitals in Ontario, Canada, while our study population was composed of infants from the greater Los Angeles area in the US [5].

Furthermore, our study did not find significant differences between infants born to families with private insurance and infants born to families with public insurance. Therefore, income differences may be mediated through insurance status. With the widespread availability of Medi-Cal, also known as Medicaid, California’s public health insurance program based on household income, families in Los Angeles have access and opportunity to affordable healthcare [20]. Moreover, the eligibility criteria for Medicaid varies by states, with California’s criteria being less restrictive compared to other states because all pregnant women residing in California are eligible for Medi-Cal coverage [21], while only pregnant women who fall under a certain income level are eligible for Medicaid in other states (i.e., Texas) [22, 23]. Medicaid eligibility differences may explain why we found no differences within our study population regarding income and insurance status; however, differences in income and insurance status may exist in other states with different Medicaid eligibility criteria. Thus, our findings support the importance of analyzing a multitude of social factors rather than focusing purely on one factor alone.

## Limitations

Several limitations are to note within this study. First, this study has a limited sample of 69 infants, only allowing us to determine sociodemographic differences/associations between our infant populations. Additionally, the limited sample size prevented us from examining causation between sociodemographic factors and the risk of CHD. The smaller sample size may also have restricted us from gaining the statistical power needed to observe statistically significant associations between other variables. However, we believe we had an adequate sample size since we detected a statistically significant association between maternal education and CHD. Second, our results may not be generalizable to other regions of the state or nation, as our study enrolled infants who were patients of CHLA and/or living in and around Los Angeles, CA, US. Third, selection bias may be of concern as mothers of higher socioeconomic status may be more likely to enroll their child as a healthy control in our original study in order to get their child’s neurodevelopmental results and may have more means of returning to follow-up visits compared to mothers of lower socioeconomic status. In addition, enrollment of infants with CHD and healthy controls may be financially motivated, as the metropolitan Los Angeles area is a predominately lower socioeconomic status area. However, it should be noted that we compensated the families with an amount that was commensurate to the amount of time spent with us during the study visits. Fourth, we did not stratify by CHD type to assess whether sociodemographic factors played a role in the severity of CHD in order to preserve our sample size. Nevertheless, our findings identified a relationship between maternal education and CHD.

## Conclusions

Our study found mothers of healthy controls to have higher levels of education compared to mothers of infants with CHD, indicating significant differences in maternal education within our research population at CHLA. We elaborated on prior studies done on CHD by proposing the possibility that maternal education may be a proxy for other sociodemographic factors. Large-scale population-based studies are needed to determine the full range of sociodemographic factors associated with the risk of CHD to help guide interventions toward lowering these risk factors in the US. If future studies find that maternal education is indeed a risk factor for CHD in the US, clinicians may use this information to identify mothers at higher risk of having an infant with CHD and refer them for further screening and follow-up. Additionally, future research may seek to answer questions about which policies and social programs may increase access and opportunity for prenatal care. These efforts can alleviate the sociodemographic differences that cause disparities in the risk of CHD.

## Figures and Tables

**Table 1.** Participants’ Sociodemographic Characteristics

Characteristic	Infants with Congenital Heart Disease N = 39 N (%)	Healthy Controls Infants N = 30 N (%)
Maternal Age (years)		
<35	25 (64.1)	13 (43.3)
≥35	14 (35.9)	17 (56.7)
Infant’s Sex		
Male	17 (43.6)	16 (53.3)
Female	22 (56.4)	14 (46.7)
Ethnicity		
Caucasian	6 (15.8)	6 (20.7)
Hispanic	26 (68.4)	14 (48.3)
Asian	4 (10.5)	2 (6.9)
African American	0 (0.0)	1 (3.5)
Other	2 (5.3)	6 (20.7)
Maternal Education		
≤2-year college/technical school	27 (75.0)	12 (40.0)
≥ 4-year college	9 (25.0)	18 (60.0)
Annual Household Income		
<$35,000	10 (33.3)	10 (34.5)
$35,000 – 74,999	12 (40.0)	5 (17.2)
≥$75,000	8 (26.7)	14 (48.3)
Total Number of People in Household		
≤3	6 (16.7)	8 (26.7)
4 – 6	26 (72.2)	20 (66.7)
≥7	4 (11.1)	2 (6.7)
Primary Language Used at Home		
English	22 (61.1)	22 (73.3)
Spanish	14 (38.9)	8 (26.7)
Insurance Status		
Public	24 (61.5)	15 (50.0)
Private	15 (38.5)	15 (50.0)

**Table 2.** Congenital Heart Defects Subtypes Among Infants with Congenital Heart Disease (N=39)

Cardiac Subtypes^a^	N (%)
**Cyanotic**	
D-TGA	8 (20.5)
HLHS	6 (15.4)
Pulmonary Atresia/IVS	1 (2.6)
SV w/Tricuspid Atresia or Stenosis	4 (10.3)
Other SV	2 (5.1)
TOF	4 (10.3)
TAPVR	3 (7.7)
Truncus Arteriosus	3 (7.7)
**Acyanotic**	
COA	6 (15.4)
Interrupted Aortic Arch	1 (2.6)
L-TGA	1 (2.6)

a D-TGA = D-Transposition of the Great Arteries; HLHS = Hypoplastic Left Heart Syndrome; IVS = Intact Ventricular Septum; SV = Single Ventricle; TOF = Tetralogy of Fallot; TAPVR = Total Anomalous Pulmonary Venous Return; COA = Coarctation of the Aorta; L-TGA = Levo-Transposition of the Great Arteries

**Table 3:** Sociodemographic Differences between Infants with Congenital Heart Disease and Healthy Controls

Characteristic	χ^2^	P-value
Maternal Age (years)	2.96	0.086
Infant’s Sex	0.65	0.422
Ethnicity	6.17	0.165
Maternal Education	8.29	0.004**
Annual Household Income	4.50	0.105
Total Number of People in Household	1.20	0.668
Primary Language Used at Home	1.10	0.294
Insurance Status	0.92	0.338

* p < 0.05;

** p < 0.01

## Data Availability

Yes.
